# The Role of Australian Mosquito Species in the Transmission of Endemic and Exotic West Nile Virus Strains

**DOI:** 10.3390/ijerph10083735

**Published:** 2013-08-19

**Authors:** Cassie C. Jansen, Scott A. Ritchie, Andrew F. van den Hurk

**Affiliations:** 1 Metro North Public Health Unit, Queensland Health, Windsor 4030, QLD, Australia; 2 School of Public Health Tropical Medicine and Rehabilitation Sciences, James Cook University, Cairns 4870, QLD, Australia; E-Mail: scott.ritchie@jcu.edu.au; 3 Public Health Virology, Forensic and Scientific Services, Department of Health, Coopers Plains 4108, QLD, Australia; E-Mail: andrew_hurk@health.qld.gov.au

**Keywords:** West Nile virus, vector, mosquito(es), Australia, Kunjin

## Abstract

Recent epidemic activity and its introduction into the Western Hemisphere have drawn attention to West Nile virus (WNV) as an international public health problem. Of particular concern has been the ability for the virus to cause outbreaks of disease in highly populated urban centers. Incrimination of Australian mosquito species is an essential component in determining the receptivity of Australia to the introduction and/or establishment of an exotic strain of WNV and can guide potential management strategies. Based on vector competence experiments and ecological studies, we suggest candidate Australian mosquito species that would most likely be involved in urban transmission of WNV, along with consideration of the endemic WNV subtype, Kunjin. We then examine the interaction of entomological factors with virological and vertebrate host factors, as well as likely mode of introduction, which may influence the potential for exotic WNV to become established and be maintained in urban transmission cycles in Australia.

## 1. Introduction

The dramatic appearance and spread of West Nile virus (WNV; family *Flaviviridae*; genus *Flavivirus*) in Northern America is a disquieting reminder of the capacity of arboviruses to establish and cause devastating public health and veterinary impacts in locations with serologically naïve populations [1]. Despite its presence in the United States for over 13 years, widespread outbreaks of WNV can still occur, as evidenced by epidemic activity in 2012 that resulted in 2,873 cases of neuroinvasive disease and 286 deaths [2]. Consequently, there has been much concern regarding the potential introduction and establishment of exotic strains of WNV in Australia, and the subsequent impacts upon human and animal health [3]. The establishment of arboviruses in new areas is certainly possible if efficient vectors, suitable vertebrate amplifying hosts and a suitable overwintering mechanism are available [4]. A range of other factors could influence potential WNV transmission in Australia, including the mode of introduction, the potential role of native fauna in transmission, suitability of the Australian climate for virus transmission, and virological factors, such as the attributes of the strain/genotype introduced and the presence of closely-related endemic arboviruses. Herein, we examine the interaction of factors that could influence the potential introduction and establishment of an exotic strain of WNV in Australia and discuss this possibility in the context of the circulation of the endemic Australian WNV Kunjin strain (WNV_KUN_). We suggest the most likely candidate species for urban transmission and present factors that would influence their roles in transmission. We conclude that given the presence of potential mosquito vectors, urban Australia could be at risk of the importation and establishment of a more pathogenic strain of WNV. 

## 2. The Threat of Exotic WNV to Urban Australia

Based on its antigenic properties, WNV is regarded as a member of the Japanese encephalitis virus serological group, which also includes Australasian members Murray Valley encephalitis (MVEV) and Japanese encephalitis viruses (JEV) [5]. There is some question regarding whether an exotic strain of WNV could establish in Australia in the presence of WNV_KUN_ [3] particularly since WNV_KUN_ is almost identical, both genetically and antigenically, to exotic strains of WNV including the New York 1999 strain (WNV_NY-99_) [6,7,8,9,10]. Attenuated WNV_KUN_ can protect against WNV_NY-99_ in mice [10] and crows [11], and it has been suggested that the presence of the endemic virus may make it more difficult for exotic WNV to spread rapidly and become established in Australia compared with what has occurred in North America [3]. However, as described by Russell and Kay [12], a viremic host or, perhaps more likely, an infected mosquito would most likely enter Australia via the eastern seaboard, where, until recently, WNV_KUN_ has seldom been active [13] and where little protective immunity would naturally exist in the vertebrate fauna or humans. Indeed, the apparent rarity of detectable flavivirus infection in urban mosquito populations in eastern Australia suggests that endemic urban flaviviruses would have minimal influence on potential exotic WNV transmission [14]. However, in 2011, a highly pathogenic strain of WNV_KUN_ caused an outbreak of acute encephalitis in horses [15,16]. Whilst centered west of the Great Dividing Range, cases were reported in coastal areas of New South Wales, suggesting that the virus can occasionally circulate near areas where an exotic strain of WNV may be introduced.

Despite WNV_KUN_ being classified as a subtype of WNV, key epidemiological differences between the viruses warrant further consideration of potential transmission cycles of exotic WNV strains. Most importantly, infection with WNV_KUN_ rarely causes disease in humans. On such rare occasions it can cause encephalitis, but is more often associated with a febrile illness that may or may not be accompanied by rash, malaise, headache, photophobia, arthralgia, myalgia and lymphadenopathy [17,18,19,20]. Beasley *et al.* [21] demonstrated that a New York strain of WNV was significantly more neuroinvasive than wild-type WNV_KUN_: 1,000–10,000 fold more infectious virus is needed to produce disease in adult mice by peripheral inoculation. Furthermore, Brault *et al.* [11] demonstrated that no illness or death occurred in WNV_KUN_-inoculated American crows compared with 100% mortality observed in those inoculated with the WNV_NY-99_ strain. Like the North American strain of WNV, WNV_KUN_ has been responsible for neurological disease in horses, although reports of severe disease in horses were rare [22] until the recent outbreak of acute encephalitis in horses in southeastern Australia [15,16].

WNV_KUN_ has been isolated from all mainland states of Australia with the majority of isolates obtained from the tropical regions of northern Western Australia, the Northern Territory, and northern Queensland, where the virus appears endemic [23,24]. WNV_KUN_ activity has been less frequently detected in the temperate regions of central and southern Australia [25]. Rather, WNV_KUN_ activity in the southeast is considered a result of the introduction from endemic northern areas, principally by migrating water birds that follow the flooding of major watercourses in the southern regions or existence in otherwise unknown cryptic foci [23,26]. Elsewhere across the globe WNV commonly circulates in urban regions. Importantly, the epidemics in southern Romania, the Volga delta in southern Russia, the northeastern USA between 1996 and 1999, and southern USA in 2012 occurred in large urban populations [1,27,28], highlighting that urban environments are at risk of potential exotic WNV introduction. In contrast, WNV_KUN_ is rarely associated with urban virus activity. 

Another key epidemiological difference between exotic WNV and WNV_KUN_ concerns vertebrate reservoir hosts. Many species of birds, particularly water birds belonging to the order Ciconiiformes, are implicated in the natural transmission cycles of WNV_KUN_, as demonstrated by field isolations and serological surveys [29,30]. Of these, laboratory studies have shown that herons and egrets may develop particularly high viremia [23,31]. Interestingly, while ardeid wading birds are important in WNV_KUN_ transmission in Australia [29,30], it seems that they are not for WNV transmission in the USA [32]. Conversely, American Crows (*Corvus brachyrhynchos*), which are considered important amplifying hosts of WNV in the USA, produce significantly lower viremia levels when infected with WNV_KUN_ than when infected with the WNV_NY-99_ strain [11]. Furthermore, unlike the strains of WNV recently circulating in the USA and Israel, there is no evidence that WNV_KUN_ is associated with disease in birds [13].

## 3. Potential Australian Vectors of West Nile Virus

A vector is considered “competent” if it permits infection, replication and transmission of a virus [33]. But, this statement does not alone indicate whether a particular species may play a role in enzootic transmission of a virus. Additional vector characteristics influence the potential role of a mosquito species in arbovirus transmission, including host feeding behavior, longevity, population densities and transmission dynamics [34]. WNV is transmitted between susceptible vertebrate hosts by mosquitoes and those belonging to the genus *Culex* are considered the primary vectors throughout its geographical range. In Africa, *Cx. univittatus* is considered to be the most important vector of WNV [35] and in Europe the principal vectors include *Cx. pipiens*, *Cx. modestus* and *Coquillettidia richiardii* [36]. Major Asian vectors are *Cx. quinquefasciatus*, *Cx. tritaeniorhynchus*, and *Cx. vishnui* [36]. The primary vectors of WNV in the USA belong to the *Cx. pipiens* complex [37,38,39]. *Cx. pipiens* and *Cx. restuans* are considered major vectors in the northeastern and north central USA [40], while *Cx. tarsalis* is particularly important in the great plains and western USA [41]. Finally, *Cx. nigripalpus* and *Cx. quinquefasciatus* are important in southeastern USA [42,43,44]. However, with the exception of *Cx. quinquefasciatus*, these mosquito species do not occur in Australia. Nevertheless, several Australian mosquito species are potential urban vectors of exotic virulent strains of WNV, based on their ability to transmit endemic flaviviruses including WNV_KUN_, host feeding behavior, urban distribution and seasonal abundance. Recent studies, involving vector competence experiments with the WNV_NY-99_ strain and analysis of host feeding patterns, have provided further evidence to incriminate candidate Australian vectors [45,46,47].

WNV_KUN_ was first isolated in 1960 from *Cx. annulirostris* at Kowanyama (previously Mitchell River Mission) in northern Queensland [48] and, since then, this species has yielded the majority of isolates [24,49,50]. It is also an efficient laboratory vector, although there is considerable intraspecific variation in transmission efficiency between populations [46,51]. WNV_KUN_ has occasionally been isolated from other Australian species including *Aedes tremulus*, *Cx. australicus*, *Cx. squamosus*, *Ae. alternans*, *Ae. normanensis*, *Ae. vigilax*, *Anopheles amictus* and *Cx. quinquefasciatus*, but the role of most of these species is likely to be secondary to *Cx. annulirostris* [24,49,50,52].

Given its accepted status as the primary vector of WNV_KUN_, it is not surprising that *Cx. annulirostris* would most likely be the primary potential vector of exotic WNV strains in Australia. This species is widely distributed on mainland Australia and has the capacity to reach high population densities under optimal weather conditions. *Cx. annulirostris* has been implicated as a major vector in urban arbovirus outbreaks [53] and is also considered to be the major vector of MVEVand JEV, which are related to WNV within the JEV serocomplex [5]. Recently, populations of *Cx. annulirostris* from eastern Australia were shown to be the most competent laboratory vectors of exotic WNV_NY-99_ from nineteen species tested, displaying transmission rates between 48% and 84% [46]. *Cx. annulirostris* is opportunistic in terms of its host feeding patterns, readily feeding on mammals and birds [45,47,54]. The percentage of mosquitoes feeding on human or birds can each exceed 20% in urban areas of Australia indicating that *Cx. annulirostris* could be involved not only in enzootic WNV transmission between birds, but also in bridge transmission to mammals, including humans.

Many WNV vectors identified abroad belong to the *Cx. pipiens* group of mosquitoes. Although Australia has four members of the *Cx. pipiens* group, including *Cx. quinquefasciatus*, *Cx. australicus*, *Cx. globocoxitus* and *Cx. molestus* [55], only *Cx. quinquefasciatus* has been assessed for its ability to become infected with and transmit WNV_NY-99_ [46]. Commensurate with its role overseas, *Cx. quinquefasciatus* was a competent vector, with at least half of the mosquitoes tested from Sydney and Brisbane expectorating virus in saliva. Although some studies have demonstrated that it does feed on humans and other mammals, *Cx. quinquefasciatus* is highly ornithophilic in urban areas of Australia [45,56,57,58] and species-specific identification of avian bloodmeals showed that 96% of avian bloodmeals identified from this species from the northern Queensland city of Cairns originated from passeriform birds [45], the primary reservoir hosts of WNV in North America [59,60,61]. For these reasons, *Cx. quinquefasciatus* has great potential not only as a primary enzootic vector facilitating virus transmission between passerine birds, but also, to a lesser extent, as a bridge vector to humans. Like *Cx. quinquefasciatus*, *Cx. australicus* is an avian feeder [56] and has yielded isolates of both WNV_KUN_ [62] and MVEV [63]. Other Australian members of the *Cx. pipiens* group, including *Cx. molestus*, should also be considered potential WNV vectors in their respective geographical distributions.

A recently introduced species into Australia [64,65], *Cx. gelidus,* is also a highly efficient laboratory vector of WNV_NY-99_ [46], as it is for a range of JEV serological group viruses, including WNV_KUN_ and JEV [66]. Whilst there is potential for this species to spread [67], *Cx. gelidus* does not currently share the wide geographical distribution of *Cx. annulirostris* or *Cx. quinquefasciatus*, being restricted mostly to focal regions in northern Australia and usually at low densities. Further, *Cx. gelidus* is largely mammalophilic [54,68] which may reduce its potential role in urban enzootic transmission of WNV. Nonetheless, the establishment of *Cx. gelidus* in Australia clearly demonstrates the potential impact of exotic mosquito species on the transmission cycles of both exotic and endemic arboviruses.

A number of other *Culex* spp. could play a regional or supplemental role in virus transmission of an exogenous WNV strain. For example, despite being a relatively poor laboratory vector of WNV _NY-99_ (transmission rates < 10%; [46]), *Cx. sitiens* readily feeds on birds [45], is widely distributed in coastal areas and can reach high population densities during favorable conditions [69]. Similarly, *Cx. squamosus* may be important for potential WNV transmission in urban centers of northern Queensland where it is abundant, has yielded isolates of WNV_KUN_ [70], and is reported to feed extensively on birds [57], supported by its abundance in the tree canopy [71].

As mentioned previously, WNV transmission is reliant upon the involvement of *Culex* mosquitoes [72,73]. Indeed, when compared with *Culex* species, Australian mosquitoes belonging to other genera are less competent for WNV_NY-99_ [46] and some species, including *An. farauti* sensu lato, appear relatively refractory to infection. Whilst flavivirus isolates are obtained from various species of *Aedes* in Australia, there is scant evidence for the involvement of these species in endemic flavivirus transmission [24]. Further, as most *Aedes* spp. are primarily mammalian feeders and typically do not feed readily on birds [45,47], *Aedes* mosquitoes are less likely than *Culex* spp. to maintain endemic WNV transmission in Australia. However, these species may be involved in transmission as bridge vectors, transmitting the virus to mammals from avian hosts, much in the same way that *Ae. albopictus* and *Ae. japonicus* do in the USA [73]. Indeed, two common Australian urban species, *Ae. notoscriptus* and *Ae. vigilax*, may fulfill the role of bridge vector for WNV, similar in the way they have been implicated in the transmission of JEV [54]. Additionally, species belonging to the genera *Aedes* or *Verrallina* with desiccation resistant eggs may be involved in virus overwintering, as they likely are for other endemic arboviruses, including the flavivirus, MVEV [74,75]. Importantly, throughout its distribution, transmission cycles of WNV are both highly variable between geographical locations and are ecologically broad, encompassing a variety of vector and host species, so the potential role of other species cannot be discounted.

## 4. Other Factors Influencing Vector Roles in WNV Transmission

We state that Australia possesses a number of mosquito species that could facilitate the establishment and maintenance of an exotic strain of WNV. Nonetheless, a number of other factors would contribute to the complexity of urban WNV transmission cycles. The impact of seasonal shifts in mosquito feeding behavior can greatly influence WNV transmission. For instance, in North America, populations of *Cx. pipiens* in the mid-Atlantic states exhibit a shift in feeding patterns from feeding predominantly on birds in early summer, to feeding moreso on humans in autumn, coinciding with the dispersal of its preferred avian host and potentially driving the observed patterns of human epidemics of WNV [76]. Unfortunately, longitudinal studies of mosquito feeding patterns in Australia are limited. Kay *et al.* [57] did not observe any seasonal patterns in host feeding behavior of *Cx. annulirostris* and *Cx. quinquefasciatus* in a remote community in northern Australia, but seasonal variation in feeding behavior has been described for *Cx. annulirostris* in restricted foci on the south coast of New South Wales [77], and may be observed in other populations and species if examined.

The implications of variability in host feeding patterns between mosquito populations can have huge implications for WNV transmission dynamics. Kramer *et al.* [1] postulates that the absence of human cases in Northern Europe, when compared with southern Europe, may be explained by differences in the feeding behavior of the dominant vector, *Cx. pipiens*. In this region, *Cx. pipiens* form molestus feeds predominantly on humans, whilst form pipiens feeds on birds. Unlike in southern Europe or in the United states, these two forms do not hybridize, so the difference in feeding behavior is distinct [38]. This phenomenon has important implications for Australia, as the taxonomy of the Australian *Cx. pipiens* group is unclear [55,78,79,80], and the associated behavioral differences between morphologically similar populations have not been fully examined. As the most widespread Australian member of this group, *Cx. quinquefasciatus,* is implicated as a major potential vector of WNV, resolution of the taxonomic status of Australian members of this group may greatly enhance the understanding of flavivirus ecology in the Australasian region.

Recently, the identification of distinct genetic lineages and, in some cases cryptic species, within *Cx. annulirostris* has provided some context for the reconsideration of both morphological and epidemiological uncertainties [81,82,83]. Indeed, further examination of population genetic structure may explain differences in vector competence observed between populations of *Cx. annulirostris* for both WNV_KUN_ [51] and WNV _NY-99_ [46]. Geographic variation in the vector competence for WNV between populations of the same mosquito species is well documented in North America [59,84,85,86]. However, behavioral differences between morphologically similar Australian vector populations have not been fully examined and are a major impediment for detailed understanding of arbovirus transmission cycles, especially if considering the potential transmission of an ecological generalist like WNV.

## 5. Vertebrate Reservoir Hosts

When incriminating mosquito species, it is also necessary to identify potential reservoir hosts which serve as a source of infection for these vectors [87,88]. A blood meal with viral titre of approximately 5 log_10_ plaque forming units (PFU)/mL is generally considered sufficient for mosquito infection [59,89,90]. In addition to physiologically supporting virus amplification, an effective reservoir for WNV must be relatively abundant in comparison with other avian species, have frequent exposure to infection via mosquitoes, and be biologically capable of infecting vector species [91]. In the USA a number of bird species, particularly those belonging to the order Passeriformes, are highly competent reservoir hosts of WNV [32,60] and, combined with high urban abundance and peridomestic distribution, are considered important for WNV ecology.

Australia possesses a rich bird diversity, which is approximately comparable in terms of species diversity to North America [92]. As WNV is considered an ecological generalist in terms of reservoir hosts, it is probable that at least some Australian bird species may fulfil the criteria required for an effective reservoir host. Only one species, the Little Raven (*Corvus mellori*) has been examined for its ability to become infected with a North American strain of WNV. After inoculation with WNV _NY-99_, *C. mellori* developed an average viremia of around 4–5 log_10_ PFU/mL, with a maximum viremia of 7 log_10_ PFU/mL in one individual [93]. Whist this viremia is only moderate if compared with that of the American crow (mean viremia up to 10.2 log_10_ PFU/mL) [60], it may be sufficient to infect at least some recipient mosquito vectors [60,90,94,95].

Clearly, there is a need to further examine the potential of other Australian bird species to amplify WNV and their degree of exposure to mosquito bites. The bird species identified from urban mosquito bloodmeal analysis [45] may suggest which bird species should be considered with priority. Other potentially important avian species that warrant examination include ardeid wading birds which are considered amplifying hosts of both WNV_KUN_ and other Australian flaviviruses [29,30,31].

There is increasing evidence that mammals and reptiles may serve as competent amplifying hosts of WNV and studies have shown that a number of small mammals are capable of developing viremias sufficient to infect mosquitoes [96,97,98,99]. Platt *et al.* [97] suggests that some of these peridomestic species, especially squirrels, may be important in WNV transmission in the urban environment in North America. Aside from birds, few vertebrate species have been implicated in JEV serological group flavivirus transmission in Australia. In addition to ardeid wading birds, pigs are primary amplifying hosts of JEV in northern Australia [100] and black flying foxes (*Pteropus alecto*) can develop a viremia sufficient to infect recipient mosquitoes [101]. If WNV were to be introduced into urban Australia it would be pertinent to consider the potential role of small peridomestic mammals such as flying foxes (*Pteropus* spp.) and marsupial possums (*Trichosurus vulpecula*; *Pseudocheirus peregrinus*) as these mammals are potential reservoirs of a number of Australian arboviruses and can be abundant in urban environments [50].

## 6. Environmental Considerations

For a virus to persist in a novel environment, it must exploit a suitable overwintering mechanism. WNV probably uses a variety of overwintering mechanisms in North America including persistent infection in mosquitoes or birds, continual low level transmission and dispersal by migratory birds [102] and, for this reason, WNV seems to be somewhat adaptive to local conditions. Accordingly, it is likely that WNV could use a range of overwintering mechanisms in various regions in Australia, depending on climate and local conditions.

Climate can affect entomological factors that impact arbovirus transmission, including host seeking behavior, the length of the extrinsic incubation period, vector activity, and the longevity of individual vectors [103,104]. The importance of temperature in the epidemiology of WNV is apparent when considering major outbreak epicenters in temperate USA, where higher than normal temperatures were always associated with virus invasion and major amplification [102]. Conversely, in warmer southern areas, average summer temperatures were adequate to initiate and maintain WNV amplification. It is possible that climatic variability across the Australian continent would likewise present divergent amplification scenarios.

Likewise, anthropogenic influences or modifications to the environment can alter the risk of arbovirus transmission, particularly in urban areas. Most recently, changes in water storage behavior in response to drought has attracted much concern due to the potential creation of urban habitats for some container-inhabiting species [105,106]. Further, the development of natural coastal wetland rehabilitation areas provides an excellent environment for both water birds and mosquito species like *Cx. annulirostris* and *Cx. sitiens* [107], potentially creating a niche for arboviruses like WNV that are maintained in a mosquito-bird transmission cycle.

## 7. Vector-Virus Interactions

The potential establishment of WNV in Australia may be driven by the strain of virus introduced. Indeed, the recent emergence of a new dominant WNV genotype (WNV_02_) in the USA has highlighted phenotypic differences between different WNV strains [108,109]. For instance, comparison between the WNV_02_ and WNV_NY-99_ strains has revealed differences in the transmission efficiency in mosquitoes [108,110], but some subsequent studies have not observed similar differences in transmission rates using the same virus strains [111]. Likewise, further phenotypic differences are evident if more divergent strains, including WNV_KUN_ are considered [11,13,15,21].

## 8. Potential Introduction of Exotic WNV

Exotic viruses may enter Australia through a range of modalities, including migratory birds, wind-blown insects and air transport of infected humans, animals and insects [3,112]. Indeed, it was proposed that wind-blown mosquitoes may have been responsible for the introduction of JEV from the New Guinea landmass into the Torres Strait and Cape York Peninsula [113]. However, introduction of wind-blown WNV-infected insects into Australia would probably be unlikely, because strains of WNV (other than WNV_KUN_) are not recognized in the Australasian region. Similarly, the introduction of WNV via a migratory bird is probably unlikely, due to both the route and duration of migration required [3].

Thus the most likely route of introduction of an exotic strain of WNV into Australia would probably be via transport in an aircraft. Of these, an infected mosquito may be the most likely threat [114]. Using a risk assessment methodology, Hernandex-Jover *et al.* [115] confirmed that infected mosquitoes arriving via aircraft arriving in Sydney represented a risk of entry into Australia, albeit considered low to moderate. Further, this study indicated that the proportion of aircraft containing infected mosquitoes and the number of mosquitoes present have a significant influence on the probability of exotic WNV entry into Australia. During a study of 307 planes arriving in Australia between 1971 and 1979, Russell *et al.* [116] determined that an average of 2.2 live mosquitoes arrived on each incoming aircraft. To best manage this threat, inflight aircraft disinsection and residual insecticidal treatments are employed [117,118].

As a number of mammals can develop viremia sufficient to infect mosquitoes [97,98,99], it is plausible that a viremic mammal, possibly a human, may be capable of importing WNV into a naïve environment. WNV is recognized as a “disease of quarantine concern” to Australia and the importation of animals is subject to strict quarantine requirements, including a period of post entry quarantine and mandatory vaccination of horses [119]. However, a viremic human or illegally imported animal may pass undetected/unregulated. Importation of human WNV infection has been documented [120], but it is unlikely that humans would develop a viremia sufficiently high to infect mosquitoes as humans typically develop only a transient viremia of low magnitude [121]. Nothwithstanding, non-arthropod-borne transmission has been documented in cases of organ transplantation, blood transfusion, intrauterine transmission and probable transmission via breast milk [122,123]. Accordingly, WNV is recognised as a potential threat to the Australian blood supply [124], and given the absence of an approved blood screening assay for WNV in Australia, donors are excluded from donating fresh blood components for four weeks after leaving an endemic country (including the USA and Canada), however plasma collection for fractionation can continue during this time [125].

As mentioned previously, exotic WNV would most likely enter Australia via the eastern seaboard [12] where, considering the rural distribution of WNV_KUN_ [13], little protective immunity from previous infection with WNV_KUN_ would be expected amongst the resident populations of potential vertebrate reservoirs [12]. Indeed, the lack of detection of any flavivirus in mosquitoes in some recent studies from urban areas of eastern Australia [14] supports the notion that previous exposure to endemic urban flaviviruses would have little impact upon the establishment of novel flaviviruses that may be introduced into these regions. Nevertheless, other endemic flaviviruses including Edge Hill and Stratford viruses have been isolated from mosquitoes collected in coastal areas of New South Wales, including some urban locations in the greater Sydney region [126].

## 9. Conclusions

Herein, we evaluated entomological factors that could influence the establishment of an exotic strain of WNV in Australia, and suggest endemic mosquito species that would most likely play a role in potential transmission cycles, particularly in urban environments. Of the common Australian mosquito species examined, *Cx. annulirostris*, *Cx. quinquefasciatus* and *Cx. gelidus* appear to be the most competent vectors and, of these, *Cx. annulirostris* also has the capacity to act as a major vector, able to facilitate both enzootic transmission and epizootic transmission from reservoir hosts to mammals. Consideration of ornithophilic feeding behavior incriminates *Cx. quinquefasciatus* as likely to be involved in enzootic transmission of WNV. Conversely, *Aedes* or *Verrallina* spp., due to observed opportunistic or mammalophilic feeding, are more likely to act only as bridge vectors that transmit the virus from birds to mammals.

From an entomological perspective, urban centers of eastern Australia could support the transmission of an exotic strain of WNV. The widespread abundance of competent vector species, in addition to evidence of host feeding on passerine bird species, suggests that the major prerequisites for transmission may be readily available. Further, the apparent rarity of flaviviruses in urban populations of mosquitoes suggests that endemic flaviviruses would have little impact on potential transmission cycles of an exotic WNV strain in urban centers. Obviously, there is a need to identify competent avian hosts in these environments, but the variety of bird species available, coupled with the knowledge that WNV can replicate in a wide variety of avian species, implies that suitable vertebrate hosts are most likely available. As has been observed in North America, it is probable that WNV transmission cycles would vary, depending on the unique environmental, climatic and ecological conditions present in different geographical locations, but hypothetical transmission in urban Australia is certainly plausible.
